# Retinopathy of prematurity shows alterations in Vegfa_164_ isoform expression

**DOI:** 10.1038/s41390-021-01646-9

**Published:** 2021-07-20

**Authors:** Olachi J. Mezu-Ndubuisi, Yong-Seok Song, Erica Macke, Hailey Johnson, Ginika Nwaba, Akihiro Ikeda, Nader Sheibani

**Affiliations:** 1 Department of Pediatrics, University of Wisconsin School of Medicine and Public Health, Madison, WI USA; 2 Department of Ophthalmology and Visual Sciences, University of Wisconsin School of Medicine and Public Health, Madison, WI USA; 3 Department of Medical Genetics, University of Wisconsin-Madison, Madison, WI USA; 4 Vanderbilt University, Nashville, TN USA; 5 Departments of Biomedical Engineering, and Cell and Regenerative Biology, University of Wisconsin School of Medicine and Public Health, Madison, WI USA

## Abstract

**Background:**

Pathologic ocular neovascularization in retinopathy of prematurity (ROP) and other proliferative retinopathies are characterized by dysregulation of vascular endothelial growth factor-A (VEGF-A). A study of Vegfa isoform expression during oxygen-induced ischemic retinopathy (OIR) may enhance our understanding of Vegf dysregulation.

**Methods:**

Following induction of OIR, immunohistochemistry and polymerase chain reaction (PCR) was performed on room air (RA) and OIR mice.

**Results:**

Total Vegfa messenger RNA (mRNA) expression was stable in RA mice, but increased in OIR mice with a peak at postnatal day 17 (P17), before returning to RA levels. Vegfa_164a_ expression was similar in both OIR and RA mice at P10 (Phase 1 OIR), but 2.4-fold higher in OIR mice compared to RA mice at P16 (Phase 2 OIR). At P10, Vegfa_164b_ mRNA was similar in OIR vs RA mice, but was expressed 2.5-fold higher in OIR mice compared to RA mice at P16. At P10 and P16, Vegfr2/Vegfr1 expression was increased in OIR mice compared to RA mice. Increased activation of microglia was seen in OIR mice.

**Conclusions:**

Vegfa_164a_, Vegfa_164b_, and Vegfr1 were overexpressed in OIR mice, leading to abnormal signaling and angiogenesis. Further studies of mechanisms of Vegf dysregulation may lead to novel therapies for ROP and other proliferative retinopathies.

**Impact:**

- Vegfa_164_ has two major isoforms, a proangiogenic, Vegfa_164a_, and an antiangiogenic, Vegfa_164b_, with opposing receptors, inhibitory Vegfr1, and stimulatory Vegfr2, but their role in OIR is unclear.
- In Phase 1 OIR, both isoforms and receptors are expressed similarly.
- In Phase 2 OIR, both isoforms are overexpressed, with an increased ratio of inhibitory Vegfr1.
- Modulation of angiogenesis by Vegf regulation enables pruning of excess angiogenesis during physiology, but results in ineffective angiogenesis during OIR. Knowledge of VEGF dysregulation may have novel therapeutic implications in the management of ROP and retinal proliferative diseases.

## Introduction

Pathological angiogenesis is a common process in several blinding eye conditions in children and adults, such as retinopathy of prematurity (ROP), proliferative diabetic retinopathy (DR), and age-related macular degeneration (ARMD). Dysregulation of vascular endothelial growth factor-A (VEGF-A) has been implicated in pathological retinal angiogenesis in these conditions,^1–3^ leading to edema, abnormal bleeding, and eventual retinal detachment, with no definitive treatment. Current treatments with laser therapy and anti-VEGF therapy for ROP,^4,5^ DR,^6,7^ and ARMD^8,9^ have limited efficacy due to concerns of toxicity.^4,5,10,11^ The specific role of VEGF-A and its isoforms and receptors in angiogenesis needs to be clarified. ROP, a major cause of neurodevelopmental disability in premature infants,^1,12,13^ has a biphasic pathogenesis with vaso-obliteration in Phase 1 and neovascularization in Phase 2, making it an ideal model^14^ to elucidate the mechanism of VEGF-A dysregulation in proliferative retinopathies.

**Fig. 1:**
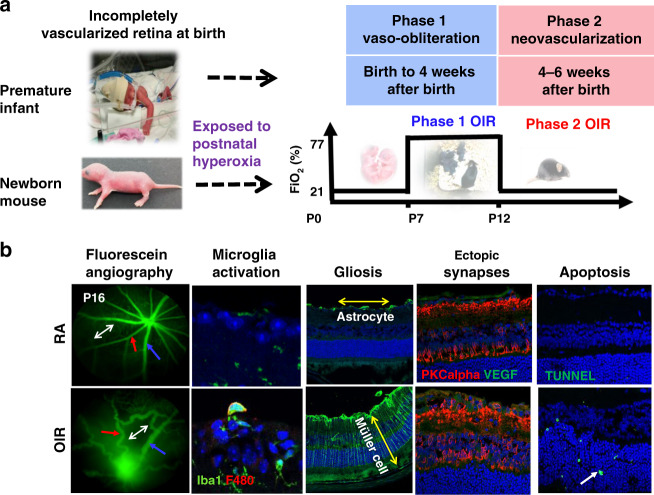
Schematic of in vivo and histologic phenotypes in ROP and OIR. **a** The newborn mouse and the premature infant both undergo postnatal retinal development. Postnatal hyperoxia triggers Phase 1 ROP (vaso-obliteration). Hypoxia leads to Phase 2 ROP (neovascularization). **b** In vivo imaging of P16 using fluorescein angiography. Blue arrows: veins; red: arteries; white double arrow: capillary network. Histology shows persistent ectopic synapses, neuronal apoptosis, and gliosis in RA and OIR mice. The yellow double arrow shows astrocytes, while the yellow double arrow shows Müller cells. The short white arrow represents TUNEL+ cells. Iba1 stains microglia. PSDS stains photoreceptor postsynaptic terminals, PKCalpha stains bipolar cells. ROP retinopathy of prematurity, TUNEL terminal deoxynucleotidyl transferase dUTP nick-end labeling, P postnatal day age.

VEGF-A is a well-known angiogenic protein, and its dysregulation in ROP and other retinal proliferative disorders,^15–17^ interrupts oxygen delivery and nutrient supply to the retina, making it insufficient to meet the high metabolic demand of the retina, thereby compromising neural function. In a study of DR using ex vivo mouse explants, VEGF-A was shown to be expressed and secreted in the retina as a neuroprotective factor when neuronal survival is threatened by adverse conditions.^18^ This infers that exposure to oxidative stress, an adverse condition, could also alter secretion of retinal VEGF-A, leading to proliferative retinopathy. In the mouse model of oxygen-induced ischemic retinopathy (OIR), newborn mice are exposed to hyperoxia from postnatal day 7 (P7) until P12, which causes retinal vessel obliteration (akin to Phase 1 ROP).^19^ From P12, they are moved to room air (RA) or relative hypoxia (similar to Phase 2 ROP) causing neovascularization. The mouse retina allows a direct window into the eye to study retinal angiogenesis. This enables evaluation of acute and long-term retinal phenotypes during developmental vascularization in live, anesthetized mice with OIR using in vivo^12,20–23^ and histological techniques.^13,24^ Fluorescein angiography (FA) reveals capillary avascularity, retinal venous dilation and arterial tortuosity, and higher capillary avascularity in neonatal OIR mice, while adult OIR mice showed variable vein dilation, persistent arterial tortuosity, and sparse capillary density despite full retinal vascularization.^12,21^ Spectral-domain optical coherence tomography demonstrates reduced retinal thickness in OIR mice compared to RA controls, which correlate negatively with the degree of capillary avascularity,^20,22^ localized to the inner retinal layer.^22^ Simultaneous in vivo studies correlate aberrant vascular and structural phenotypes in neonatal and adult OIR mice to reduce the inner abnormal retinal function in neonatal and adult OIR mice using electroretinogram,^23^ and histological studies showing prolonged cellular apoptosis, Müller cell gliosis, microglia activation, and ectopic synapse formation^25^ (Fig. 1). However, it is unclear how precisely the dysregulation of VEGF expression affects the abnormal OIR recovery and phenotype of insufficient angiogenesis in adult OIR mice.

VEGF-A_165_, the most predominant VEGF-A isoform in the eye, is similar to mouse Vegfa_164_, which is one amino acid residue shorter than the human VEGF-A_165_. Studies have explored therapies to promote retinal VEGF-A messenger RNA (mRNA) and protein expression to ameliorate vaso-obliteration. Use of retinoic acid during Phase I was noted to stabilize VEGF-A mRNA levels and reduce vaso-obliteration in OIR mice, and attenuate later excessive generation of VEGF-A in Phase 2, thereby preventing hypoxia-induced retinal neovascularization in OIR mice.^16^ In our prior study, exogenous administration of VEGF-A_165a_ in intravitreal microparticle form P13 (a day after hyperoxia exposure) promoted earlier revascularization in OIR at P20, but we found that total Vegfa levels measured at P13 were increased 6-fold in OIR mice compared to age-matched RA mice,^1^ prior to exogenous VEGF-A_165a_ administration. This suggests the possibility that excessively high endogenous VEGF-A could render the protein functionally inactive, and may represent varying proportions of pro- and antiangiogenic splice variants. VEGF-A_165_ isoforms have two splice variants: proangiogenic VEGF-A_165a_ and antiangiogenic VEGF-A_165b_.^26^ The antiangiogenic splice variants of VEGF-A, VEGF-A_xxxb,_ are shown to inhibit VEGF’s stimulation of endothelial proliferation, migration, and vasodilatation.^27^ However, the specific distribution and roles of these isoforms in pathologic retinopathy and OIR need further study.

Here, we show that there is a differential expression and distribution of Vegfa_164_ isoform splice variants and Vegf receptors in Phase 1 and 2 OIR. We found a 2.4-fold higher Vegfa_164a_ expression and a 2.5-fold higher Vegfa_164b_ expression in OIR mice compared to RA mice in Phase 2 OIR. Vegfr1 was noted to be upregulated in OIR mice. It is unclear if this is due to VEGF-A_164a_ suppression from autocrine signaling, predominant action of VEGF-A_164b_ protein activity, or altered isoform interaction with Vegf receptors. In OIR mice, we show in this study that an increase in Vegf expression is associated with a disruption of synaptic integrity, as well as activation of macrophages. Understanding the mechanism of VEGF-A isoform and receptor interactions and their association with microglia activation may shed more light on their effect on retinal phenotypes, and help identify novel therapeutic targets for proliferative retinopathies.

## Methods

### Animals

Wild-type C57BL/6J mice (Jackson Laboratory, Bar Harbor, ME) were reared under approved protocols by the Institutional Animal Care and Use Committee of the University of Wisconsin School of Medicine and Public Health, and in compliance with the Association for Research in Vision and Ophthalmology Statement for the Use of Animals in Ophthalmic and Vision Research. At P7, OIR mice were exposed to 77 ± 2% oxygen in a chamber (Biospherix ProOX 110; Apex Lab, Redwing, MN) with nursing dams for 5 days, while age-matched mice were raised in RA, as previously described in a published modification^28^ of an established mouse OIR protocol.^19^ All applicable international, national, and/or institutional ethical guidelines for the care and use of animals were met.

### Immunohistochemistry

Histological studies were performed at P19, P24, P32, and P47 to represent each phase of retinal vascular development, early (P16–20), mid (P23–27), late (P30–34), and mature (beyond P34), as previously published.^28^ Eyes were fixed in 4% paraformaldehyde for 2 h at 4 °C and then cryoprotected at 4 °C in a graded series of sucrose. Eyes were embedded in optimal cutting temperature compound (Sakura Finetek, Torrance, CA) and sectioned at 12 μm thickness. For immunohistochemistry on cryostat sections, sections were air-dried for 2 h and blocked in phosphate-buffered saline (PBS) with 0.5% Triton X-100 and 2% normal donkey serum for 1 h at room temperature. Next, sections were incubated overnight with the primary antibodies against the molecule of interest. Vegf (R&D Systems, AB-293-NA) and protein kinase C-alpha (PKCalpha, Sigma, P4334) staining were done to detect total Vegf expression and determine synaptic integrity of rod bipolar cells, respectively. Ionized calcium-binding adaptor molecule 1 (Iba1, Wako, 019-19741) and F4/80 (Abcam, ab6640) cells were stained to determine the distribution and character of microglia in the retina. F4/80 stains macrophages of bone marrow origin, while Iba1 stains resident macrophages in the retina. Sections were then rinsed in PBS and incubated with a 1:200 diluted Alexa 488-conjugated secondary antibody (Thermo Scientific, Rockford, IL) and/or Cy3-conjugated secondary antibody (Jackson ImmunoResearch Laboratories, West Grove, PA) for 45 min at room temperature. Sections were imaged on a Zeiss 510 confocal laser scanning system using ZEN software (Carl Zeiss Microimaging) or on a Leica SP8 stimulated emission depletion 3× microscope (Leica Microsystems, Germany). F4/80+ macrophages were quantified from a 100 μm square retinal section of 200 μm from the center of the retina for each condition and time point (*n* = 3). VEGF+ areas were quantified using the threshold function of ImageJ 1.46r and represented as % green area of the total area measured, which were calculated for each condition and time point (*n* = 3). ImageJ reference: NIH Image to ImageJ: 25 years of image analysis.^29^

### Quantitative polymerase chain reaction (qPCR)

Mice were enucleated after retinal imaging, and retinas dissected and snap frozen in liquid nitrogen. Total RNA was extracted from homogenized mouse tissues using a combination of TRIzol (Life Technologies, Grand Island, NY) reagent and RNeasy mini kit (Qiagen, Valencia, CA) following the manufacturer’s instructions. Complementary DNA (cDNA) was synthesized using RNA to cDNA EcoDry Premix kit (Clontech, Mountain View, CA) according to the manufacturer’s instructions. Quantitative real-time PCR (qRT-PCR) was performed in a total volume of 20 µL containing ten of TB-Green Advantage qPCR Premix (Clontech). qRT-PCR was performed to determine mean mRNA expression of total Vegfa at random time points in RA (P7, P14, P21, and P42) and OIR (P12, P15, P17, and P18) mice. Then, in a separate experiment, qRT-PCR was done to determine mean mRNA expression of Vegfa_164a_, Vegfa_164b_, Vegfr1, and Vegfr2 at P10 (peak of hyperoxia or Phase 1 OIR), P13 (early Phase 2), and P16 (peak of neovascularization or Phase 2 OIR). Multiple time points in Phase 2 OIR (beyond P16) were not studied because they are not expected to show any variation in Vegf expression since the phenotype of OIR in Phase 2 is consistently one of excess angiogenesis. The expression levels of target genes were normalized to the housekeeping gene *Rpl13a*. The qPCR primer sequences were as follows: Vegfa164a forward, 5′-GCAGCTTGAGTTAAACGAACG-3′ and Vegfa164a reverse, 5′-GGTTCCCGAAACCCTGAG-3′; Vegfa164b forward, 5′-GTTTGTCCAAGATCCGCAGAC-3′ and Vegfa164b reverse, 5′-GAGAGGTCTGCAAGTACGTTC-3′; Vegfr1 forward, 5′-GGCCCGGGATATTTATAAGAAC-3′ and Vegfr1 reverse, 5′-CCATCCATTTTAGGGGAAGTC-3′; Vegfr2 forward, 5′-CCCCAAATTCCATTATGACAAC-3′ and Vegfr2 reverse, 5′-CGGCTCTTTCGCTTACTGT-3′; Rpl13a forward, 5′‐TCTCAAGGTTGTTCGGCTGAA‐3′ and Rpl13a reverse, 5′‐CCAGACGCCCCAGGTA‐3′.

### Statistical analysis

Statistical analyses were performed in GraphPad Prism 6 (GraphPad Software, La Jolla, CA). The statistical difference between groups was calculated using unpaired Student’s two-tailed *t* test, and analysis of variance using the GraphPad Prism software. *P* < 0.05 was considered to be statistically significant, using *P < 0.05, **P < 0.01, ***P < 0.001, and ****P < 0.0001. All data are presented as the mean ± the standard deviation (SD).

## Results

### Similar VEGF-A expression in both RA and OIR mice

**Fig. 2:**
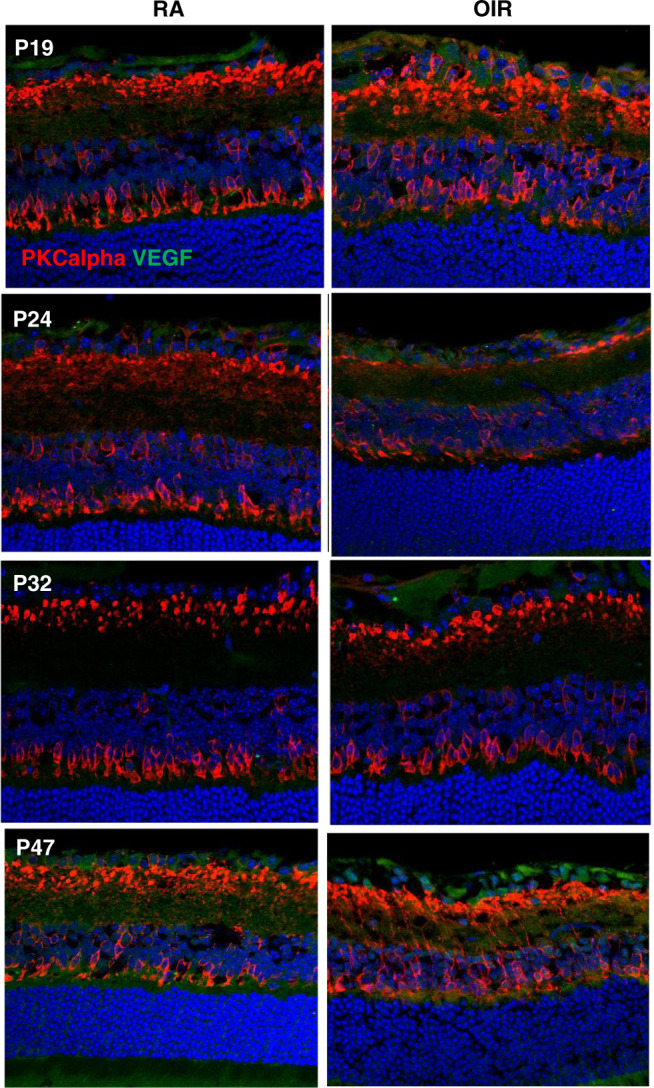
VEGF-A_165_ expression and synaptic integrity in RA and OIR. Vegfa_165_ antibody (green) staining was observed to be increased in both RA (*n* = 4) and OIR (*n* = 4) mice at P19. While some retinal areas in OIR mice appeared to have increased VEGF-A expression, there was no difference between total VEGF-A_164_ expression in RA and OIR mice at any age. PKCalpha (red) showed mis-aligned rod bipolar cells in OIR mice, while RA mice had uniformly aligned synapses. Vegfa_165_ vascular endothelial growth factor-a_164_, RA room air, OIR oxygen-induced retinopathy, P postnatal day age.

To study Vegfa expression during OIR, retinal cross-sections were stained with VEGF-A_165_ antibody (Fig. 2). The VEGF-A_165_ antibody staining was likely identifying the expression of both Vegfa_164a_ and Vegfa_164b_ isoforms in the retina. While several retinal areas in OIR mice appeared to have increased VEGF-A expression, the quantification of VEGF-A_164_ in immunohistochemistry cross-sections showed that there was no difference between total VEGF-A_164_ expression in RA and OIR mice at any age, P19 (5.4 ± 2.62, *n* = 3 vs 5.41 ± 0.49, *n* = 3, *P* > 0.05), P24 (4.85 ± 0.54, *n* = 3 vs 9.79 ± 4.05, *n* = 3, *P* > 0.05), P32 (2.84 ± 1.84, *n* = 3 vs 3.44 ± 0.33, *n* = 3, *P* > 0.05), and P47 (4.66 ± 2.46, *n* = 3 vs 4.07 ± 1.74, *n* = 3, *P* > 0.05). PKCalpha showed mis-aligned rod bipolar cells in OIR mice, indicating loss of synaptic integrity. RA mice had uniformly aligned rod bipolar cells.

### Increased activated macrophages in OIR mice

**Fig. 3:**
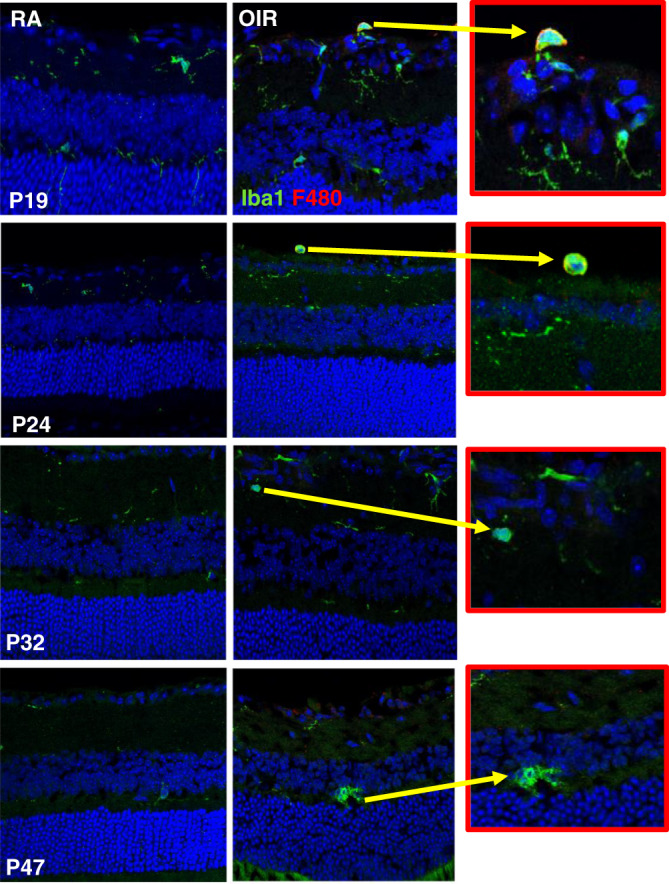
Microglia activation in RA and OIR mice. Two subsets of Iba1+ cells were noted in OIR mice: ramified, dendritic-appearing and amoeboid, round-appearing. Certain microglia subset co-stained both Iba1+ and F4/80+. Ameoboid, rounded F4/80+ microglia represented <5% of microglia. RA mice had sparse number of microglia, mainly in a ramified, dendritic phenotype, typical of resting microglia. In OIR mice, Iba1+ microglia was increased, highest at P19, and decreased with age. F4/80 staining was increased at P19 and P24 in OIR mice compared to RA mice, but similar at P32 and P47 in RA and OIR mice. Iba1-stained cells were higher in OIR compared to RA mice at every age tested. The ratio of F4/80 and Iba1 cells was increased at P24, with 30% higher proliferation of Iba1 cells. Yellow arrow shows a magnified view of activated amoeboid microglia in OIR mice that co-stained both Iba1+ and F4/80+. RA room air, OIR oxygen-induced retinopathy, P postnatal day age.

Two distinct subsets of Iba1+ cells were noted in OIR mice: ramified, dendritic-appearing and amoeboid, round-appearing. Certain microglia subsets co-stained for both Iba1 and F4/80. Amoeboid, rounded F4/80+ microglia represented <5% of microglia, and were only seen in OIR mice. RA mice had a sparse number of microglia, mainly in a ramified, dendritic phenotype, typical of resting microglia. In OIR mice, Iba1+ microglia were increased compared to RA mice at P19 (17.33 ± 4.16, *n* = 3 vs 7.00 ± 1.73, *n* = 3, *P* = 0.02), P24 (11.67 ± 3.79, *n* = 3 vs 5.33 ± 1.53, *n* = 3, *P* = 0.05), P32 (16.33 ± 2.52, *n* = 3 vs 6.67 ± 2.52, *n* = 3, *P* = 0.009) and P47 (7.67 ± 0.58, *n* = 3 vs 3.67 ± 1.53, *n* = 3, *P* = 0.013). The Iba1+ cell proliferation was highest at P19, and decreased with increasing postnatal day age (Fig. 3). Quantification of F4/80+ microglia showed a higher proliferation in OIR compared to RA mice at P19 (3.33 ± 2.08, *n* = 3 vs 0.0 ± 0.00, *n* = 3, *P* = 0.05) and P24 (3.67 ± 1.15, *n* = 3 vs 0.0 ± 0.00, *n* = 3, *P* = 0.005), but similar numbers at P32 (2.33 ± 1.53, *n* = 3 vs 0.33 ± 0.58, *n* = 3, *P* = 0.1) and P47 (1.33 ± 2.30, *n* = 3 vs 0.00 ± 0.00, *n* = 3, *P* = 0.38). When the ratio of F4/80+ and Iba1+ microglia were evaluated, there was no difference between this ratio in RA and OIR at all ages, except at P24 where the F4/80+/Iba1+ microglia ratio was 30% higher in OIR compared to RA mice (0.32 ± 0.0.08, *n* = 3 vs 0.0 ± 0.00, *n* = 3, *P* < 0.0001).

### Vegfa_164_ expression peaked at P17 in OIR mice

**Fig. 4:**
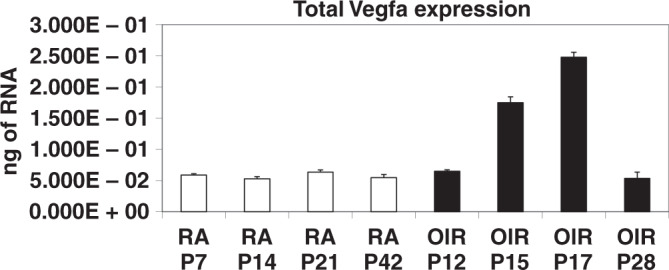
Total Vegfa_164_ expression in RA and OIR mice. In RA mice, total Vegfa_164_ mRNA expression was stable and unchanged throughout postnatal retinal vascular development. In OIR mice, there was an increase in total VEGF-A_164_ expression postnatally with a peak at P15 to P17, and a decrease to basal levels by P28. Vegfa_164_ vascular endothelial growth factor-a_164_, RA room air, OIR oxygen-induced retinopathy, P postnatal day age. Values are stated as mean ± SD.

Using qRT-PCR, we randomly surveyed the total Vegfa_164_ expression at different time points throughout retinal vascular development to look at both Vegfa isoforms together during normal retinal vascular development and OIR. During retinal vascular development, P7 would represent the time point of complete formation of superficial retinal vascular plexus. In OIR mice, P7 is the time-point when hyperoxia exposure is started. P14 would represent the time point when the superficial and deep retinal vascular plexus is formed. P21 time-point represents the time point of formation of all three retinal vascular layers (superficial, intermediate, and deep plexus). P42 represents mature retinal vasculature and a time-point where there is ongoing vascular remodeling to regulate angiogenesis. We showed that the expression of total Vegfa was stable in RA mice when tested at P7 (5.891E − 02 ± 2.088E − 03 ng, *n* = 3), P14 (5.266E − 02 ± 3.520E − 03 ng, *n* = 3), P21 (6.341E − 02 ± 3.536E − 03 ng, *n* = 3), and P42 (5.457E − 02 ± 5.157E − 03 ng, *n* = 3). However, in OIR mice there was an increase in Vegfa expression from P12 (6.500E − 02 ± 2.262E − 03 ng, *n* = 3) to P15 (1.748E − 01 ± 9.406E − 03 ng, *n* = 3), with a peak at P17 (2.476E − 01 ± 8.062E − 03 ng, *n* = 3), and a sharp decline to basal levels at P28 (5.354E − 02 ± 9.905E − 03 ng, *n* = 3) (Fig. 4). The expression of Vegfa at P28 appeared comparable to the levels seen at P14, P21, and P42 in RA mice. RA mice showed an even expression of total Vegfa throughout retinal vascular development, while OIR mice showed a brief peak at P19 prior to decline to RA levels.

### Vegfa_164a_ and Vegfa_164b_ expression were similar in OIR and RA mice in Phase 1 OIR

**Fig. 5:**
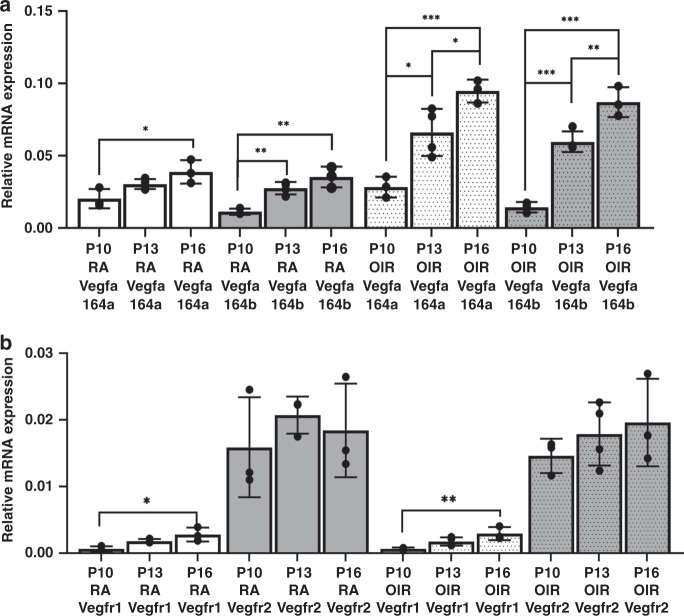
Mean mRNA expression of Vegfa_164a_ and Vegfa_164b_ and Vegf receptors 1 and 2 in RA and OIR mice at P10, P13, and P16. **a** Comparing mean mRNA expression of Vegfa_164a_ and Vegfa_164b_ between RA and OIR mice. At P10, there was no difference in the expression of Vegfa_164a_ in OIR vs RA mice (0.028 ± 0.007 vs 0.020 ± 0.007, *P* = 0.23, *n* = 3), and no difference in Vegfa_164b_ expression in OIR vs RA mice (0.014 ± 0.004 vs 0.011 ± 0.002, *P* = 0.27). At P16, Vegfa_164a_ was 2.4-fold higher in OIR mice compared to RA mice (0.095 ± 0.008 vs 0.038 ± 0.008, *P* = 0.001, *n* = 3), while Vegfa_164b_ was expressed 2.5-fold higher in OIR mice compared to RA mice (0.087 ± 0.01 vs 0.035 ± 0.007, *P* = 0.002). In RA mice, Vegfa_164a_ was significantly higher at P16 compared to P10 (*P* = 0.017), but remained unchanged between P10 and P13 (*P* = 0.14) and between P13 and P16 (*P* = 0.23). Vegfa_164b_ increased from P10 to P13 (*P* = 0.008), remained unchanged from P13 to P16 (*P* = 0.17), and was higher at P16 compared to P10 (*P* = 0.001). **b** Comparing mean mRNA expression of Vegfr1 and Vegfr2 between RA and OIR mice In RA mice, Vegfr1 did not change from P10 to P13 (*P* = 0.16) and from P13 to P16 (*P* = 0.25), but increased at p16 compared from P10 (*P* = 0.018). In RA mice, Vegfr2 did not change from P10 to P13 (*P* = 0.63) or from P13 to P16 (*P* = 0.89), and there was no difference between P10 and P16 (*P* = 0.87). In OIR mice, Vegfr1 was unchanged at P10 and P13 (*P* = 0.14) and from P13 to P16 (*P* = 0.12), but was significantly higher at P16 compared to P10 (*P* = 0.01). However, Vegfr2 remained unchanged from P10 to P13 (*P* = 0.67), from P13 to P16 (*P* = 0.89), and was similar at P16 compared to P10 (*P* = 0.46). Vegfr vascular endothelial growth factor receptor, RA room air, OIR oxygen-induced retinopathy, P postnatal day age. RA (*n* = 3), and OIR (*n* = 3) for each age. Significance is set at *P* < 0.05, *P < 0.05, **P < 0.01 ***P < 0.001. Values are stated as mean ± SD.

To determine the distribution of proangiogenic Vegfa_164a_ and antiangiogenic Vegfa_164b_ in Phase 1 OIR, their mean expression was evaluated using qRT-PCR at P10 (Fig. 5a). During OIR, mice are exposed to hyperoxia from P7 to P12, and P10 is the peak of hyperoxia exposure. At P10, there was no difference in the expression of Vegfa_164a_ in OIR vs RA mice (0.028 ± 0.007 vs 0.020 ± 0.007, *P* = 0.23, *n* = 3). At P10, there was no difference in the expression of Vegfa_164b_ in OIR vs RA mice (0.014 ± 0.004 vs 0.011 ± 0.002, *P* = 0.27, *n* = 3). At P10, there was no difference in the Vegfa_164a_/Vegfa_164b_ expression in RA mice (*P* = 0.08), while in OIR mice Vegfa_164a_, it was 2-fold higher than Vegfa_164b_ expression (*P* = 0.0399).

### Both Vegfa_164a_ and Vegfa_164b_ were higher in OIR compared to RA mice in Phase 2 OIR

The mean mRNA expression of Vegfa_164a_ and Vegfa_164b_ were assessed during Phase 2 OIR by performing qRT-PCR at P16 (Fig. 5a). During OIR, the mice are returned to RA at P12 after 5 days of hyperoxia, and then experience relative hypoxia with the resumption of neovascularization. The phenotype at P16 is predominantly avascular in the central retina, which is devoid of capillaries, while the major retinal vessels are formed. We found that at P16, Vegfa_164a_ was 2.4-fold higher in OIR than RA mice (0.095 ± 0.008 vs 0.038 ± 0.008, *P* = 0.001, *n* = 3). while Vegfa_164b_ was expressed 2.5-fold higher in OIR mice than RA mice (0.087 ± 0.01 vs 0.035 ± 0.007, *P* = 0.002). Vegfa_164a_ increased 2.0-fold at P16 compared to P10 in RA mice (*P* = 0.03), but in OIR mice had a 3.4-fold increase from P10 to P16 (*P* = 0.0004). Vegfa_164b_ expression increased 3.2-fold at P16 compared to P10 in RA mice during physiologic vascularization (*P* = 0.005), and increased 6.2-fold at P16 compared to P10 in OIR mice (*P* = 0.0003). Therefore, at P16, Vegfa_164a_ expression was 1.6 times higher in OIR mice than RA mice (*P* = 0.001), while Vegfa_164b_ expression was 1.9 times higher at P16 in OIR mice compared to RA mice (*P* = 0.002).

### At P13 (transitional phase), Vegfa_164a_ and Vegfa_164b_ remained stable in RA mice, but increased in OIR mice

In RA mice, Vegfa_164a_ expression remained stable from P10 to P13 (0.02 ± 0.007, *n* = 3 vs 0.03 ± 0.003, *n* = 4, *P* = 0.14), and remained stable between P13 and P16 (*P* = 0.23), and was significantly higher at P16 compared to P10 (*P* = 0.0173). In RA mice, Vegfa_164b_ expression increased from P10 to P13 (0.011 ± 0.002, *n* = 3 vs 0.0275 ± 0.004, *n* = 4, *P* = 0.008), remained stable between P13 and P16 (*P* = 0.166), and was significantly higher at P16 than P10 (*P* = 0.001). In OIR mice, Vegfa_164a_ expression increased from P10 to P13 (0.028 ± 0.007, *n* = 3 vs 0.066 ± 0.02, *n* = 4, *P* = 0.011), and increased again from P13 to P16 (0.066 ± 0.016, *n* = 4 vs 0.09 ± 0.008, *n* = 3, *P* = 0.041), and was significantly higher at P16 than P10 (*P* = 0.0007). In OIR mice, *Vegfa*_*164b*_ expression increased from P10 to P13 (*P* = 0.011), and increased significantly from P13 to P16 (*P* < 0.0001), and was significantly higher at P16 than P10 (*P* = 0.005) (Fig. 5a).

### Vegfr1 expression was higher in Phase 2 compared to Phase 1 in both RA and OIR mice

To determine if the differential expression of Vegfa isoforms affected their interactions with Vegf receptors, the mean mRNA expression of Vegfr1 and Vegfr2 were assessed by performing qRT-PCR at P10 (Phase 1 OIR) and P16 (Phase 2 OIR). At P10, Vegfr1 expression was equivalent in both OIR and RA mice (0.00065 ± 0.00017 vs 0.00067 ± 0.00030, *P* = 0.93, *n* = 3) (Fig. 5b). At P16, Vegfr1 expression was equivalent in both OIR and RA mice (0.0029 ± 0.00079 vs 0.0028 ± 0.00084, *P* = 0.86, *n* = 3). Going from P10 to P16, Vegfr1 was expressed 4.2-fold higher in RA mice (*P* = 0.028), and 4.5-fold higher in OIR mice (*P* = 0.016).

### Vegfr2 was similar in RA and OIR mice in Phase 1 and 2 OIR

Vegfr2 was equivalently expressed in both RA and OIR mice in Phase 1 and 2 OIR. At P10, Vegfr2 expression was similar in OIR and RA mice (0.014 ± 0.002 vs 0.016 ± 0.006, *P* = 0.79, *n* = 3). At P16, Vegfr2 expression was equivalent in both OIR and RA mice (0.02 ± 0.005 vs 0.018 ± 0.006, *P* = 0.84, *n* = 3). There was no difference in Vegfr2 expression going from P10 to P16 in RA (*P* = 0.69) and OIR mice (*P* = 0.29) (Fig. 5b).

### The ratio of Vegfr2/Vegfr1 decreased from Phase 1 to Phase 2 OIR, but remained equivalent in RA and OIR mice

At P10, Vegfr2 expression was 24-fold higher compared to Vegfr1 expression in RA mice (*P* = 0.025), while Vegfr2 expression was 22-fold higher compared to Vegfr1 expression in OIR mice (*P* = 0.0007). At P16, the relative expression of Vegfr2 decreased in both RA and OIR mice, such that Vegfr2 was expressed 6.6-fold higher compared to Vegfr1 in RA mice (*P* = 0.019). Conversely, Vegfr2 was expressed 6.7-fold higher compared to Vegfr1 in OIR mice (*P* = 0.012). Therefore, there was a greater increase in Vegfr1 going from P10 to P16 in OIR mice compared to RA mice.

### At P13 (transitional phase), Vegfr1 and Vegfr2 remained stable in RA mice, but increased in OIR mice

In RA mice, Vegfr1 expression remained stable from P10 to P13 (0.0007 ± 0.0004, *n* = 3 vs 0.002 ± 0.0003, *n* = 4, *P* = 0.1614), and remained stable between P13 to P16 (0.002 ± 0.0003, *n* = 4 vs 0.003 ± 0.001, *n* = 3, *P* = 0.25), and was significantly higher at P16 compared to P10 (*P* = 0.019). In RA mice, Vegfr2 expression was unchanged from P10 to P13 (0.02 ± 0.008, *n* = 3 vs 0.02 ± 0.003, *n* = 4, *P* = 0.63), remained stable between P13 to P16 (*P* = 0.89), and again from P10 than P16 (*P* = 0.87). In OIR mice, Vegfr1 expression was unchanged from P10 to P13 (0.0007 ± 0.0002, *n* = 3 vs 0.002 ± 0.0006, *n* = 4, *P* = 0.14), and remained stable from P13 to P16 (0.002 ± 0.0006, *n* = 4 vs 0.002 ± 0.001, *n* = 3, *P* = 0.12), but was significantly higher at P16 compared to P10 (*P* = 0.01). In OIR mice, Vegfr2 expression was unchanged from P10 to P13 (0.015 ± 0.003, *n* = 3 vs 0.018 ± 0.005, *n* = 4, *P* = 0.67) and remained stable from P13 to P16 (0.02 ± 0.005, *n* = 4 vs 0.02 ± 0.007, *n* = 3, *P* = 0.89), and again was unchanged from at P16 than P10 (*P* = 0.46) (Fig. 5b).

## Discussion

ROP and other severe sight-threatening retinal proliferative disorders in infants and adults like ROP, DR, and AMD are characterized by disordered angiogenesis and dysregulation of the angiogenic factor, VEGF-A. Unfortunately, anti-VEGF therapies have met with limited success due to associated adverse effects. The role of VEGF-A isoform and its pro- and antiangiogenic splice variants during angiogenesis has not been fully elucidated and may contribute to pathologic retinopathies. Using an OIR mouse model to study retinal angiogenesis, we have previously shown abnormal vascular recovery following oxidative stress with histological evidence of prolonged activation of microglia, Müller cell gliosis, ectopic synapses, and apoptosis. We and others showed that exogenous therapies to increase VEGF-A levels promote revascularization of vaso-obliterated retinal areas,^1,16^ despite our finding of higher than normal endogenous Vegfa levels in OIR mice compared to RA mice.^1^ The contribution of Vegf isoforms and receptors to angiogenesis during OIR is not completely understood. In this study, we revealed that Vegfa_164_ splice variants are differentially expressed during OIR, with overexpression of both proangiogenic Vegfa_164a_ and antiangiogenic Vegfa_164b_ above basal levels along with upregulation of Vegfr1 more than Vegfr2 in Phase 2 OIR. Further studies of the mechanisms of Vegfa ligand/receptor interactions during angiogenic signaling may enhance understanding of the pathologic neovascularization, and aid in the development of safe and effective therapies for retinal proliferative diseases.

### Overexpression of Vegfa_164a_ and Vegfa_164b_ in Phase 2 OIR

In this study, although the quantification of total Vegf expression did not vary differently between RA and OIR mice in retinal cross-sections, our investigation of Vegf isoform splice variants showed a difference in expression of the mRNA of proangiogenic Vegfa_164a_ and antiangiogenic Vegfa_164b_ isoform splice variants at P10 (Phase 1), P13 (Early Phase 2), and P16 (Phase 2) OIR. We observed that there was no difference in the expression of either isoform in RA or OIR mice during Phase 1 OIR in hyperoxia (P7–P12). However, during Phase 2 OIR in room air or relative hypoxia (after P12), there was a 2-fold increase in Vegfa_164a_ in RA mice and a 3.4-fold increase in Vegfa_164a_ in OIR mice. On the other hand, Vegfa_164b_ increased 3.2-fold in RA mice and 6.2-fold in OIR mice going from P10 to P16. Vegfa_164a_ and Vegfa_164b_ increased from P10 to P16 in both RA and OIR mice, but in RA mice both isoforms remained stable from P13 to P16. However, in RA mice, from P10 to P13, Vegfa_164a_ remained stable, while Vegfa_164b_ increased. Therefore, there was an antiangiogenic shift in RA mice at P13. These findings suggest that basal levels of both proangiogenic Vegfa_165a_ and antiangiogenic Vegfa_165b_ are secreted early in postnatal life to maintain a physiologic balance that allows normal retinal vascularization in RA mice, but is limited by hyperoxia exposure in OIR mice allowing vascularization by major blood vessels and inhibition of capillary vascularization. The overexpression of both Vegfa_164a_ and Vegfa_164b_ isoform splice variants in Phase 2 in our current study was modest in RA mice allowing physiologic neovascularization with precise vascular pruning or remodeling to prevent excess neovascularization, but was excessive in OIR mice leading to abnormal angiogenesis with arterial tortuosity, venous dilation, and insufficient capillary density despite peripheral vascularization, as shown in our previous studies.^21^ This suggests an autocrine regulation of angiogenesis when Vegfa is overexpressed, resulting in a negative feedback signaling to limit angiogenesis in both RA and OIR mice, albeit dysregulated in OIR mice. A recent study showing that the migration of human umbilical vein endothelial cells towards VEGF when treated with higher concentrations of VEGF (1 μg/mL) was significantly less than when treated with lower concentrations of VEGF (0.025, 0.1, and 0.5 μg/mL)^30^ supports our findings of negative angiogenic effects during excess VEGF levels. Our study is in agreement with the findings of Boudria and colleagues that VEGF_165b_ stimulates the proliferation and invasion of lung tumor cells in an autocrine manner.^31^ It is unclear if the abnormal OIR phenotype is a manifestation of inhibition of Vegfa_164a_ action or an upregulation of Vegfa_164b_ action on Vegfa receptors.

### Vegfr1 was significantly higher in Phase 2 compared to Phase 1 in both RA and OIR mice

We next studied the expression of Vegf receptors in OIR. VEGF-A binds to its tyrosine kinase membrane-bound receptors, VEGF-R1 (Flt-1) and VEGF-R2 (Flk-1/KDR), to promote the activation of downstream signaling pathways that control proliferation, survival, and migration of endothelial cells during neovascularization.^3,32–37^ VEGF-R1 is shown to inhibit angiogenesis, while VEGF-R2 is known to stimulate angiogenesis,^38^ but their exact roles in OIR are largely unknown. We found that Vegfr1 was expressed 4.2-fold higher in RA mice and 4.5-fold higher in OIR mice at P16 compared to P10. Vegfr2 was similar in RA and OIR mice in both Phase 1 and 2 OIR, with no difference in expression between P10 and P16. At P10, Vegfr2 expression was 24-fold higher than Vegfr1 in RA mice, while Vegfr2 expression was 22-fold higher than Vegfr1 in OIR mice. At P16, Vegfr2 expression decreased in both RA and OIR mice, but was 6.6-fold higher than Vegfr1 in RA mice and 6.7-fold higher than Vegfr1 in OIR mice. Vegfr1 and Vegfr2 were unchanged in expression at P10, P13, and P16 in RA mice. However, in OIR mice, Vegfr1 and Vegfr2 were overexpressed at P16 compared to P10, but remained stable during the transitional phase from P10 to P13 and from P13 to P16. This indicates a decrease in angiogenic signaling in RA mice with the maturation of retinal vascularization, which allows vascular remodeling while preventing excess signaling. However, OIR mice appear to have a higher overall increase in Vegfr1 expression going from P10 to P16, leading to inefficient angiogenesis. Our findings are supported by a study showing a linear increase in VEGF-R1 mRNA expression 60-fold higher at P26 than at P3, with the ratio of VEGF-R2 mRNA to VEGF-R1 mRNA expression ranging from 200-fold at P3 to two-fold at P26.^39^ In an in vitro study of adult mouse subventricular zone neural progenitors, a high dose (500 ng/mL) of VEGF significantly downregulated endogenous VEGF receptors 1 and 2, which significantly reduced neural progenitor cell proliferation and enhancement of neuronal differentiation, while a low dose (50 ng/mL) of VEGF significantly upregulated endogenous VEGF-R1 and VEGF-R2, but did not increase proliferation and differentiation, suggesting that exogenous VEGF-A has a biphasic effect on the expression of endogenous VEGF receptors.^40^ We speculate that during OIR, excess Vegfa ligand based on the microenvironment can stimulate or inhibit angiogenic activity by preferential interaction with either the proangiogenic signaling receptor Vegfr2 or the angio-inhibitory Vegfr1 receptor. Our findings are in agreement with a study showing that excess VEGF-A following choroidal neovascularization incited by injury activated VEGF-R1 and deactivated VEGF-R2 resulting in antiangiogenic effects in a dose-dependent manner,^41^ and a study showing that VEGF-R1 suppresses the excessive development of hemangioblasts,^42^ by trapping or sequestering excess VEGF in order to regulate the amount of available free VEGF for vascular development, such that the absence of VEGF-R1 leads to dysregulation in the amount of untrapped VEGF resulting in excessive development of the endothelial lineage.^43^ The mechanisms of Vegf ligand and Vegf receptor interactions warrant further exploration.

### Aberrant Vegf signaling during OIR may drive abnormal angiogenesis and long-term OIR phenotype

The specificity in secretion and differential action of VEGF-A isoforms and receptors in OIR may allude to problems with altered regulation of VEGF expression, ineffective protein activity, or abnormal receptor interaction, which cause the abnormal OIR long-term phenotype. Our study suggests that the retina is sensitive to the precise level of VEGF-A_164_ in hypoxia following hyperoxic injury, such that if levels exceed pre-hyperoxic injury levels, VEGF-R1 signaling predominates in the retinal microenvironment following excess VEGF-A, compared to the normal developing retina without hyperoxia where VEGF-R2 is dominantly expressed. A study by Gerber et al. showing that hypoxia selectively upregulates VEGF-R1 without upregulating VEGF-R2 supports our findings.^44^ The effective decrease in the ratio of Vegfr2/Vegfr1 or the upregulation of Vegfr1 in Phase 2 OIR appears to be a physiologic phenomenon of vascular pruning to regulate angiogenesis and prevent excessive neovascularization in RA mice. In OIR mice, the mechanism of upregulation of Vegfr1 may be altered due to overexpression of the Vegfa_164_ ligand isoforms in OIR mice, leading to ineffective angiogenesis from their negative interaction with Vegfr2 receptor. Vegfr1 acts to soak up excess Vegfa_164_ and competes with Vegfr2 to inhibit or negatively modulate angiogenesis. This is consistent with the phenotype of capillary sparsity in mature OIR mice with the absence of neovascular buds unlike the dense capillary networks of OIR mice with neovascular buds, representing insufficient angiogenesis. Our prior studies showed gliosis of Müller cells, microglia activation, and formation of ectopic synapses between photoreceptor cells and bipolar cells in OIR mice, persisting in adulthood (Fig. 1). Our current studies revealed a robust numerical proliferation of microglia in OIR more than RA mice at every age tested. We also showed an increase in F4/80+ macrophages at P19 and P24 when measured 200 μm from the center of the retina in OIR mice compared to RA, indicating microglia activation. P24 was the only time point that showed a difference in the ratio of F4/80 and Iba1 proliferation, with iba1-stained microglia being 30% more than F4/80-stained microglia. The glial cells in the retina provide support and a cross-talk between neurons and vascular cells. Retinal glial cells consist of macroglia, such as astrocytes and Müller cells, and microglia, the primary resident immune cells of the retina, which become activated quickly after an injury.^45^ In pathologic conditions, Müller cells undergo reactive proliferation or hyperplasia to protect the retina from further damage,^46,47^ as shown in our previous studies.^25^ Müller cells and macrophages are the main producers of VEGF in the retina.^48,49^ When *VEGF* gene is deleted in Müller cells, ischemia-induced retinal angiogenesis and vascular leakage are repressed, inferring that Müller cell-secreted VEGF is a major contributor to retinal angiogenesis.^49^ In addition to playing a critical role in synaptogenesis, retinal microglia have been shown to be major sources of VEGF.^50^ In OIR, it is unclear whether the predominant source of VEGF that is being overexpressed is from microglia or Müller cell. Ectopic synapses seen in our retinal cross-sections were associated with increased VEGF expression, suggesting a contributory role from VEGF dysregulation. In our current studies, two subsets of microglia are observed—resting and activated microglia (Fig. 3). The origins of retinal microglia remain a source of debate, and may be key to determining the character and function of microglia during OIR. Oxidative stress or exogenous VEGF has been shown to induce a significant increase of VEGF mRNA, which was abolished by inhibition of VEGF-R2 in an autocrine manner.^15^ Müller cells appear to release basal levels of VEGF through autocrine signaling as an antiapoptotic factor essential for the maintenance of Müller cell survival and viability.^51–53^ The relationship between VEGF isoforms/receptors and macrophage/glia function need further investigation and was beyond the scope of this paper.

### Implications for therapeutic application of abnormal Vegf signaling

In earlier published studies, we successfully administered intravitreal VEGF-A_165a_ microparticles at P13 in OIR mice, which resulted in earlier revascularization of the OIR retina at P20, despite high circulating levels of total Vegfa, which represents both a and b splice variants.^1^ Our recent studies lend a new understanding that the mechanism of exogenous proangiogenic VEGF-A success in OIR mice is likely related to overcoming the altered VEGF signaling. P13 represents early Phase 2 OIR that has a phenotype of residual vaso-obliteration and ongoing neovascularization, and shows unique transitions in Vegfa_164a_ *and Vegfa*_*164b*_ expression as elucidated in our study. Injecting exogenous proangiogenic VEGF-A_165a_ microparticles likely enhances the angiogenic effect in the retina by saturation of Vegf receptors, specifically overcoming the inhibitory activity of Vegfr1. Vegfr1 competes with Vegfr2, thereby administering exogenous VEGF provides more ligands available to activate Vegfr2 by saturating Vegfr1, so it cannot effectively compete with Vegfr2. Unopposed Vegfr2 signaling upregulates angiogenic signaling pathways. It is therefore plausible clinically to utilize exogenous proangiogenic Vegfa_165a_ microparticles to promote angiogenesis or earlier recovery from vaso-obliteration in infants with ROP. Further studies of mechanistic signaling of Vegfa ligand and receptors are mandated prior to translational considerations.

## Conclusion

In summary, ROP and other sight-threatening retinal proliferative disorders are characterized by VEGF dysregulation. ROP’s biphasic pathogenesis allows studies of the intricate actions of VEGF in the retina during hyperoxia (Phase 1) and hypoxia (Phase 2), making the mouse an excellent model for OIR due to its postnatal retinal vascularization. We showed that VEGF isoforms are differentially expressed during OIR, with overexpression of the angiogenic isoform, Vegfa_164a_ in Phase 2 OIR, and increased expression of the inhibitory Vegfr1 over Vegfr2, leading to abnormal and ineffective vascularization in OIR mice. Aberrant angiogenic signaling in OIR mice may be related to ineffective Vegfr2 receptor inactivity, the negative autocrine effect of overexpressed Vegfa_164_, and preferential angiogenic signaling from VEGF-producing cells, like macrophages and Müller cells. Our findings indicate a critical cross-talk between vascular cells, neuronal cells, and glia cells in the early and late stages of the proliferative retinopathies. Investigating mechanisms of Vegf dysregulation may enable the identification of novel therapeutic targets for ROP and other proliferative retinopathies.

## References

[1] Mezu-Ndubuisi, O. J. et al. Intravitreal delivery of VEGF-A165-loaded PLGA microparticles reduces retinal vaso-obliteration in an in vivo mouse model of retinopathy of prematurity. *Curr. Eye Res.* **364**, 275–286 (2019). 10.1080/02713683.2018.1542736 PMC6661072 30383455

[2] Cruz-Guilloty, F. et al. 2013 Infiltration of proinflammatory m1 macrophages into the outer retina precedes damage in a mouse model of age-related macular degeneration. *Int. J. Inflamm.* **2013**, 503725 (2013). 10.1155/2013/503725 PMC3606733 23533946

[3] Ye X (2012). ERK1/2 signaling pathway in the release of VEGF from Muller cells in diabetes. Invest. Ophthalmol. Vis. Sci..

[4] Mintz-Hittner HA, Kennedy KA, Chuang AZ (2011). Efficacy of intravitreal bevacizumab for stage 3+ retinopathy of prematurity. N. Engl. J. Med..

[5] Good WV (2004). Final results of the early treatment for retinopathy of prematurity (ETROP) randomized trial. Trans. Am. Ophthalmol. Soc..

[6] Michaelides M (2010). A prospective randomized trial of intravitreal bevacizumab or laser therapy in the management of diabetic macular edema (BOLT study): 12-month data: report 2. Ophthalmology.

[7] Distefano, L. N, Garcia-Arumi, J., Martinez-Castillo, V. & Boixadera, A. Combination of anti-VEGF and laser photocoagulation for diabetic macular edema: a review. *J. Ophthalmol.* **2017**, 2407037 (2017). 10.1155/2017/2407037 PMC5350536 28348882

[8] Guymer RH (2019). Subthreshold nanosecond laser intervention in age-related macular degeneration: the LEAD randomized controlled clinical trial. Ophthalmology.

[9] Gemenetzi M, Lotery A, Patel P (2017). Risk of geographic atrophy in age-related macular degeneration patients treated with intravitreal anti-VEGF agents. Eye.

[10] Wu L (2008). Twelve-month safety of intravitreal injections of bevacizumab (Avastin): results of the Pan-American Collaborative Retina Study Group (PACORES). Graefes Arch. Clin. Exp. Ophthalmol..

[11] Victor, A. A. et al. Intravitreal bevacizumab in diabetic macular edema at RSUP Cipto Mangunkusumo in 2017. *Int. J. Retina* **2**, (2019).

[12] Mezu-Ndubuisi, O. J. et al. In-vivo retinal vascular oxygen tension imaging and fluorescein angiography in the mouse model of oxygen-induced retinopathy. *Invest. Ophthalmol. Vis. Sci.* **54**, 6968–6972 (2013). 10.1167/iovs.13-12126 PMC3808101 24052641

[13] Stahl A (2009). Computer-aided quantification of retinal neovascularization. Angiogenesis.

[14] Mezu-Ndubuisi OJ (2013). In vivo retinal vascular oxygen tension imaging and fluorescein angiography in the mouse model of oxygen-induced retinopathy. Invest. Ophthalmol. Vis. Sci..

[15] Rossino MG (2020). Oxidative stress induces a VEGF autocrine loop in the retina: relevance for diabetic retinopathy. Cells.

[16] Wang L (2014). Up-regulation of VEGF by retinoic acid during hyperoxia prevents retinal neovascularization and retinopathy. Invest. Ophthalmol. Vis. Sci..

[17] Hellstrom A (2001). Low IGF-I suppresses VEGF-survival signaling in retinal endothelial cells: direct correlation with clinical retinopathy of prematurity. Proc. Natl Acad. Sci. USA.

[18] Amato R, Biagioni M, Cammalleri M, Dal Monte M, Casini G (2016). VEGF as a survival factor in ex vivo models of early diabetic retinopathy. Investig. Ophthalmol. Vis. Sci..

[19] Smith LE (1994). Oxygen-induced retinopathy in the mouse. Investig. Ophthalmol. Vis. Sci..

[20] Mezu-Ndubuisi OJ (2014). Correspondence of retinal thinning and vasculopathy in mice with oxygen-induced retinopathy. Exp. Eye Res..

[21] Mezu-Ndubuisi OJ (2016). In vivo angiography quantifies oxygen-induced retinopathy vascular recovery. Optom. Vis. Sci..

[22] Mezu-Ndubuisi OJ, Taylor LK, Schoephoerster JA (2017). Simultaneous fluorescein angiography and spectral domain optical coherence tomography correlate retinal thickness changes to vascular abnormalities in an in vivo mouse model of retinopathy of prematurity. J. Ophthalmol..

[23] Mezu-Ndubuisi, O. J., Adams, T., Taylor, L. K., Nwaba, A. & Eickhoff, J. Simultaneous assessment of aberrant retinal vascularization, thickness, and function in an in vivo mouse oxygen-induced retinopathy model. *Eye* **195**, 363–373 (2019). 10.1038/s41433-018-0205-1 PMC6460691 30209267

[24] Sheibani, N. et al. Inhibition of retinal neovascularization by a PEDF-derived nonapeptide in newborn mice subjected to oxygen-induced ischemic retinopathy. *Exp. Eye Res.* **195**, 108030 (2020). 10.1016/j.exer.2020.108030 PMC7282953 32272114

[25] Mezu-Ndubuisi OJ (2020). Long-term evaluation of retinal morphology and function in a mouse model of oxygen-induced retinopathy. Mol. Vis..

[26] Peach CJ (2018). Molecular pharmacology of VEGF-A isoforms: binding and signalling at VEGFR2. Int. J. Mol. Sci..

[27] Manetti M (2011). Overexpression of VEGF165b, an inhibitory splice variant of vascular endothelial growth factor, leads to insufficient angiogenesis in patients with systemic sclerosis. Circ. Res..

[28] Mezu-Ndubuisi OJ (2016). In vivo angiography quantifies oxygen-induced retinopathy vascular recovery. Optom. Vis. Sci..

[29] Schneider, C. A., Rasband, W. S. & Eliceiri, K. W. NIH Image to ImageJ: 25 years of image analysis. *Nat. Methods* **9**, 671–675 (2012). 10.1038/nmeth.2089 PMC5554542 22930834

[30] Morita, A. et al. The process of revascularization in the neonatal mouse retina following short-term blockade of vascular endothelial growth factor receptors. *Cell Tissue Res.* **382** 529–549 (2020). 10.1007/s00441-020-03276-9 32897421

[31] Boudria A (2019). VEGF 165b, a splice variant of VEGF-A, promotes lung tumor progression and escape from anti-angiogenic therapies through a β1 integrin/VEGFR autocrine loop. Oncogene.

[32] Ablonczy Z, Crosson CE (2007). VEGF modulation of retinal pigment epithelium resistance. Exp. Eye Res..

[33] Gu X (2003). Hyperoxia induces retinal vascular endothelial cell apoptosis through formation of peroxynitrite. Am. J. Physiol..

[34] Wang H (2015). IGF-1 signaling via the PI3K/Akt pathway confers neuroprotection in human retinal pigment epithelial cells exposed to sodium nitroprusside insult. J. Mol. Neurosci..

[35] Vessey K, Wilkinson Berka J, Fletcher E (2011). Characterization of retinal function and glial cell response in a mouse model of oxygen induced retinopathy. J. Comp. Neurol..

[36] Nishi J (2008). Vascular endothelial growth factor receptor-1 regulates postnatal angiogenesis through inhibition of the excessive activation of Akt. Circ. Res..

[37] Ferrara N, Gerber H-P, LeCouter J (2003). The biology of VEGF and its receptors. Nat. Med..

[38] Meyer RD, Mohammadi M, Rahimi N (2006). A single amino acid substitution in the activation loop defines the decoy characteristic of VEGFR-1/FLT-1. J. Biol. Chem..

[39] Shih S-C, Ju M, Liu N, Smith LE (2003). Selective stimulation of VEGFR-1 prevents oxygen-induced retinal vascular degeneration in retinopathy of prematurity. The. J. Clin. Investig..

[40] Meng H (2006). Biphasic effects of exogenous VEGF on VEGF expression of adult neural progenitors. Neurosci. Lett..

[41] Nozaki M (2006). Loss of SPARC-mediated VEGFR-1 suppression after injury reveals a novel antiangiogenic activity of VEGF-A. The. J. Clin. Investig..

[42] Fong G-H, Zhang L, Bryce D-M, Peng J (1999). Increased hemangioblast commitment, not vascular disorganization, is the primary defect in flt-1 knock-out mice. Development.

[43] Hiratsuka S, Minowa O, Kuno J, Noda T, Shibuya M (1998). Flt-1 lacking the tyrosine kinase domain is sufficient for normal development and angiogenesis in mice. Proc. Natl Acad. Sci. USA.

[44] Gerber H-P, Condorelli F, Park J, Ferrara N (1997). Differential transcriptional regulation of the two vascular endothelial growth factor receptor genes Flt-1, but not Flk-1/KDR, is up-regulated by hypoxia. J. Biol. Chem..

[45] Chen L, Yang P, Kijlstra A (2002). Distribution, markers, and functions of retinal microglia. Ocul. Immunol. Inflamm..

[46] Faktorovich EG, Steinberg RH, Yasumura D, Matthes MT, LaVail MM (1990). Photoreceptor degeneration in inherited retinal dystrophy delayed by basic fibroblast growth factor. Nature.

[47] Fletcher EL (2010). The significance of neuronal and glial cell changes in the rat retina during oxygen-induced retinopathy. Doc. Ophthalmol..

[48] Lajko M (2016). Hyperoxia-induced proliferative retinopathy: early interruption of retinal vascular development with severe and irreversible neurovascular disruption. PLoS ONE.

[49] Bai Y (2009). Müller cell‐derived VEGF is a significant contributor to retinal neovascularization. J. Pathol..

[50] Rathnasamy G, Foulds WS, Ling E-A, Kaur C (2019). Retinal microglia—a key player in healthy and diseased retina. Prog. Neurobiol..

[51] Saint-Geniez M (2008). Endogenous VEGF is required for visual function: evidence for a survival role on Müller cells and photoreceptors. PLoS ONE.

[52] Grigsby J (2017). Autocrine and paracrine secretion of vascular endothelial growth factor in the pre-hypoxic diabetic retina. Curr. Diabetes Rev..

[53] Matsuda M (2017). Cellular stress response in human Müller cells (MIO-M1) after bevacizumab treatment. Exp. Eye Res..

